# A case of acute appendicitis due to intestinal schistosomiasis

**DOI:** 10.1016/j.amsu.2018.11.015

**Published:** 2018-11-26

**Authors:** Mugahid A. Salih

**Affiliations:** University of Khartoum, Faculty of Medicine, Sudan

**Keywords:** Appendicitis, Schistosomiasis

## Abstract

**Introduction:**

Intestinal Schistosomiasis is a parasitic disease caused by *Schistosoma mansoni* or *Schistosoma japonica.* Sudan is considered one of the endemic areas of schistosomiasis and many studies were done as regard to the prevalence and impact of the disease. Schistosomal appendicitis is rare, particularly in developed countries compared to endemic areas.

**Case report:**

This is a case of 36 –year- old man with past history of schistosomiasis presented with features of intestinal obstruction to Ibrahim Malik teaching hospital in Khartoum-Sudan. Exploratory laparotomy revealed gangrenous appendix. Histopathology results came back as extensive necrosis of the appendix with multiple viable Schistosomal ova. Stool analysis revealed no ova. The patient received praziquantel therapy and now he is in a good condition.

**Conclusion:**

Clinicians and pathologist should be aware of schistosomal appendicitis, particularly in endemic areas like Sudan.

## Highlights

• Schistomiasis can have variable ways of clinical presentations especially in endemic countries.
• Acute appendicitis can be one of the clinical presentations of schistosoma.
• Clinicians and pathologists should be aware of this rare presentation of schistosoma.

## 1 Introduction and literature review

This work has been reported in line with the SCARE criteria [1]^.^

Intestinal Schistosomiasis is a parasitic disease caused by *Schistosoma mansoni* or *Schistosoma japonica*, and is transmitted mainly through contact with water sources [2]. Schistosomal appendicitis is very rare, particularlyin developed countries like the USA, Europe, and Japan [2]. A review of 311 pathologic archival specimens of vermiform appendix over a period of 10 years in Japan revealed only one case of schistosomal appendicitis [2]. This is in contrast to the prevalence of this presentation in endemic areas such as Africa and South Asia [2]. Sudan is considered one of the endemic areas of schistosomiasis and many studies were done as regard to the prevalence and impact of the disease [3,4]^.^ However, there is few reported cases of intestinal Schistosomiasis presenting as acute appendicitis in Sudan. In the literature, Schistosoma haematobium was reported as the inducer of a granulomatous inflammatory response in addition to eosinophilia and fibrosis of the appendix. Oviposition was associated with frank acute appendicitis, and serosal involvement resulting in peritoneal adhesions and ileoileal intussusception [5]. An African American retrospective review of 1690 appendicectomies reported three cases of Schistosomal appendicitis with transmural inflammation predominantly with neutrophils and scanty eosinophils. Schistosomal granulations were present in all the layers of appendix including the serosa [6]. Many similar studies were mentioned in the literature [7]. In one study, the involvement of the vermiform appendix by schistosomiasis was found in (4.2%) of all the appendicectomy specimens, with the majority having histologically proven acute appendicitis [8]. The role of schistosomes in the pathogenesis of acute appendicitis was investigated in other studies and its generally thought that there are two types of schistosomal appendicitis, obstructive and granulomatous [9].

## 2 Case report

**Fig. 1:**
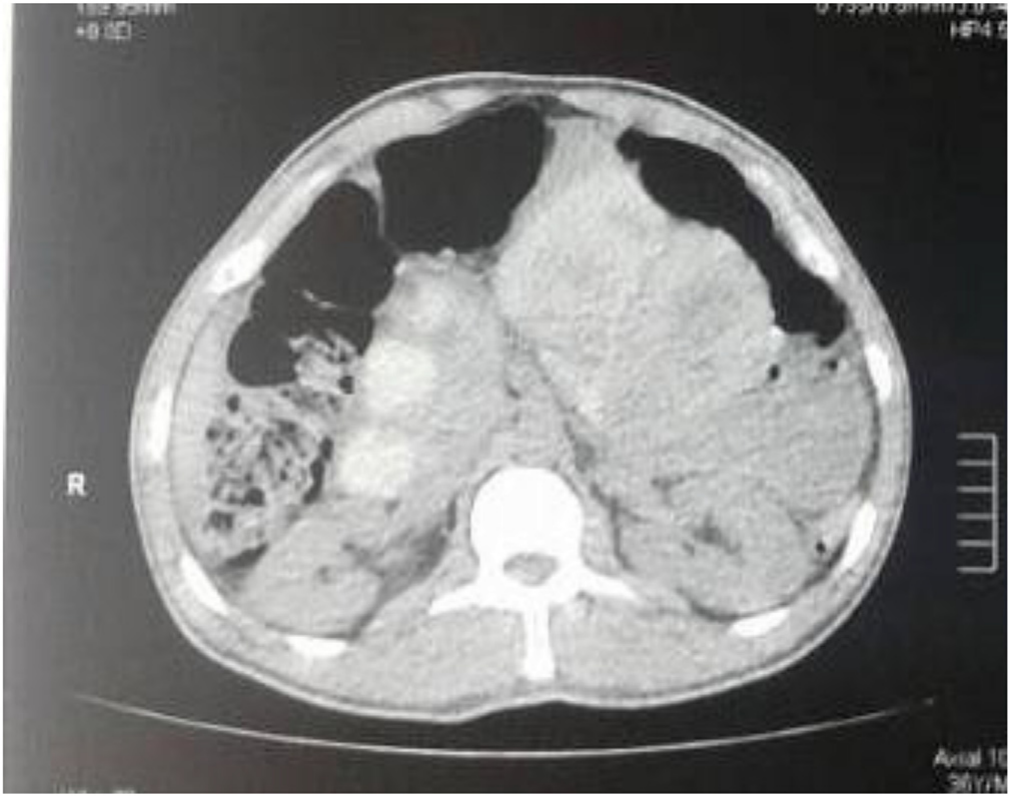
CT scan of the abdomen showing dilated bowel loops and inflammatory mass on the right iliac fossa.

**Fig. 2:**
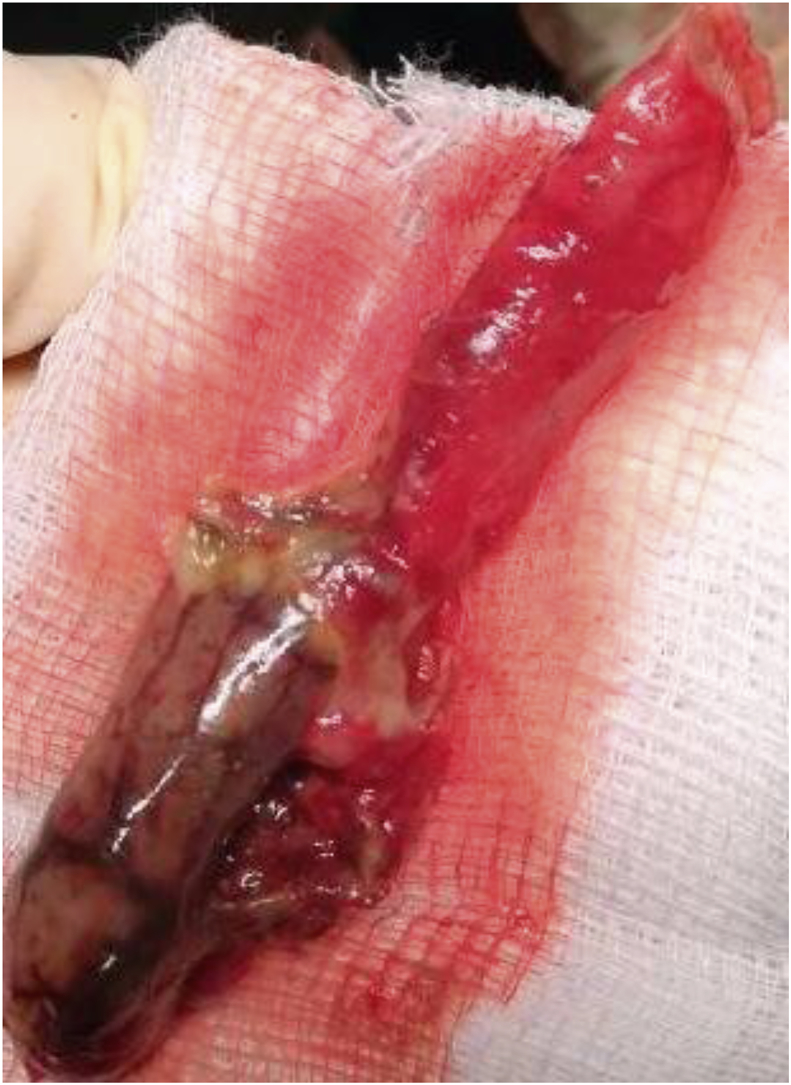
The gangrenous appendix after removal.

**Fig. 3:**
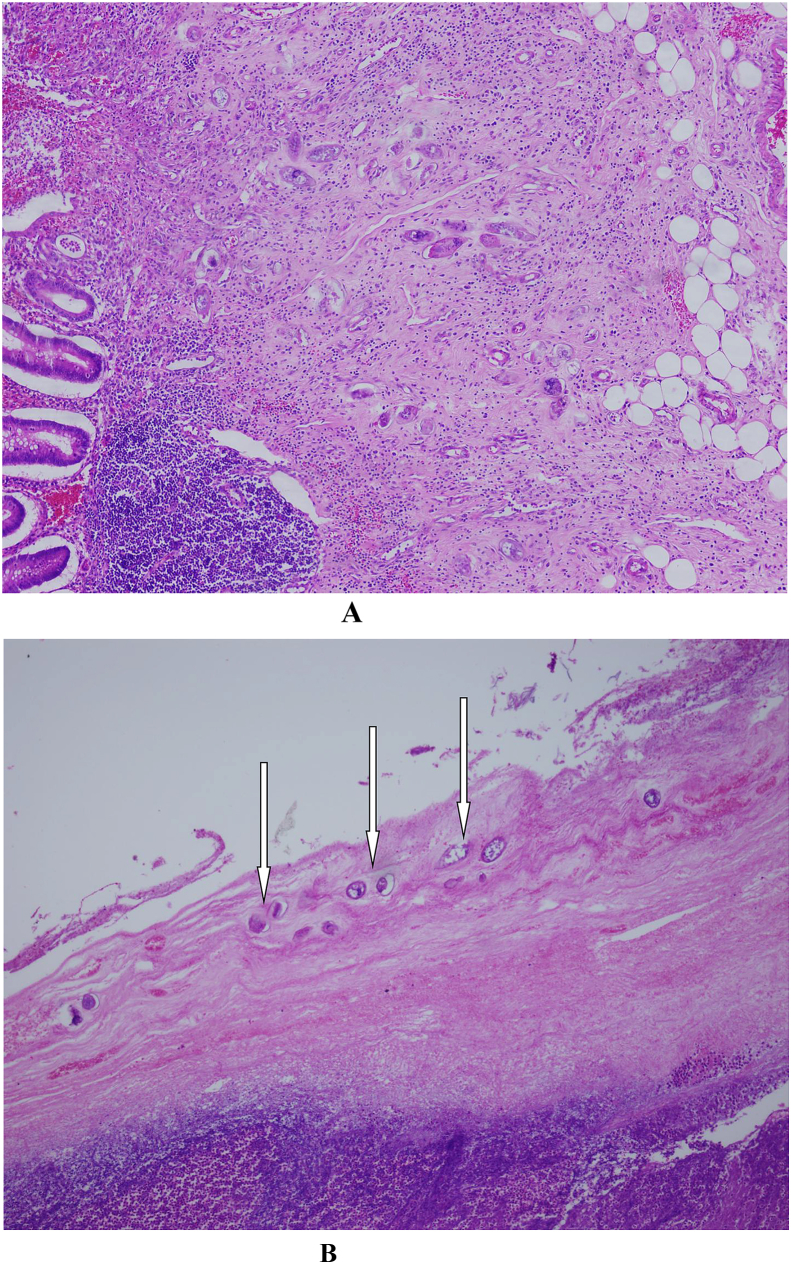
**A** and **B** histopathological section of the inflamed appendix with the arrows pointing to the schistosomal ova.

This is a case of 36-year-old man who presented with features of intestinal obstruction to our institution. He was diagnosed in Egypt as a case of intestinal Schistosomiasis one year prior to admission and received treatment. However, follow up investigation to confirm eradication of the disease was not done. The patient presented to our surgical department complaining of absolute constipation, abdominal pain, abdominal distention and vomiting. On examination the abdomen was distended and bowel sounds were absent. There was tenderness in the lower part of the abdomen and digital rectal examination revealed collapsed rectum. Computed tomography (**CT**) scan of the abdomen revealed dilated bowel loops (Fig. 1). The patient was resuscitated adequately and exploratory laparotomy was done through infra-umbilical midline incision. There was localized turbid fluid collection, dilated small and large bowel loops due to paralytic ileus and gangrenous appendix. Appendectomy was done (Fig. 2) followed by lavage and closure. The patient received antibiotics and passed through a smooth postoperative period and was discharged home after one week approximately. The appendix specimen was sent for histopathology and the result came back as extensive necrosis of the appendix with multiple viable Schistosomal ova (Fig. 3). Subsequent stool analysis revealed no ova. Despite of that the patient received a single dose of praziquantel therapy and he is quite well now.

## 3 Discussion

Schistosomiasis is a parasitic disease transmitted through contact with contaminated water sources. It is related to bad personal hygiene and environmental sanitation. This explains the dominance of this disease in the endemic areas within the developing countries. The disease is endemic in Sudan [3,4]. Schistosoma mansoni and Schistosoma japonica represents the types which infest the intestine. Their main complications are hepatointestinal, however the involvement of the appendix is rare and few cases were reported [[5], [6], [7], [8], [9]], in particular from developed countries [2].

In fact, the predominance of features of intestinal obstruction in the present case is considered to be an atypical presentation for acute appendicitis. Such way of presentation has been rarely reported and it might be linked to paralytic ileus.

The diagnosis of Schistosomal appendicitis in this case was confirmed using reliable histopathological methods. Despite the negativity of stool analysis for Schistosomal ova, there is an evidence of past history of Shistosomiasis which strongly support the current diagnosis.

It is now agreed that appendiceal schistosomiasis may be the atiology of acute appendicitis. This may be due to ischemic changes caused by intravascular egg impaction. Moreover, this may diminish mucosal immunity, increasing the risk of bacterial infection [2].

In endemic areas like Nigeria, Badmos et al. [6] reported that appendices with schistosomiasis were present in 35/843 (4.2%) of surgically resected cases. Of these 35 positive cases, 23 (65.7%) were associated with acute appendicitis, while the remaining 12 cases (34.3%) were not. Thus, the presence of the parasite does not always give rise to acute appendicitis.

## 4 Conclusion

Clinicians and pathologist should be aware of schistosomal appendicitis, particularly in endemic areas like Sudan.

## Ethical approval

The research was ethically approved.

## Sources of funding

The case report was not funded.

## Author contribution

The author Mugahid A Salih IS a general surgeon who met the case and he is the one who wrote the manuscript.

## Conflicts of interest

No conflict of interest.

## Registration of research studies

None.

## Guarantor

The guarantor is the author Mugahid A Salih.

## Patient consent

The patient signed informed consent.

## Provenance and peer review

Not commissioned, externally peer reviewed.
